# Supplementary material for: Construction of Mechanically Reinforced Thermoplastic Polyurethane from Carbon Dioxide-Based Poly(ether carbonate) Polyols via Coordination Cross-Linking
Source: Polymers (Basel). 2021 Aug 17;13(16):2765. doi: 10.3390/polym13162765 (PMC8399931; doi:10.3390/polym13162765)
Supplement: Supplementary file 1 [file polymers-13-02765-s001.zip › polymers-1324023-supplementary.pdf]

polymers

Supplementary Materials: Construction of Mechanically Reinforced Thermoplastic Polyurethane from Carbon Dioxide-Based Poly(ether carbonate) Polyols via Coordination Cross-Linking

Gaosheng Gu, Jincheng Dong, Zhongyu Duan and Binyuan Liu *

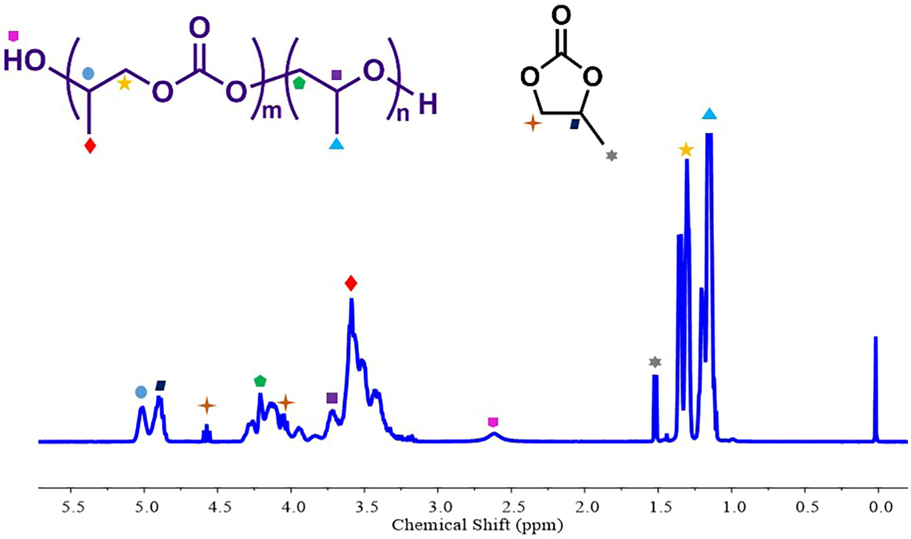

Figure S1. ^{1}H NMR spectrum of PPCD.

Table S1. Data summary of PPCD.

Sample	M_n [a] (g/mol)	$Đ$ [a]	C [b] (mg KOH/g)	OHV [b] (mg KOH/g)	CU [c] (%)
PPCD	2.2×10^3	1.43	0.1	53.9	31.2

[a] Determined from GPC; [b] *C*: acid value, *OHV*: hydroxyl value, determined by titration; [d] *CU*: carbon dioxide content, calculated from ^{1}H NMR spectrum with the formula of $CU = A_{5.0} + A_{4.2} - 2A_{4.58} / (A_{5.0} + A_{4.2} - A_{4.58} + A_{3.5}) \times 100\%$, in which the A_x means the integral area in chemical shift x; In the preparation of PU, using Corrected *OHV* (Corrected $OHV = OHV + C$) to calculate the feeding ratio of TPU.

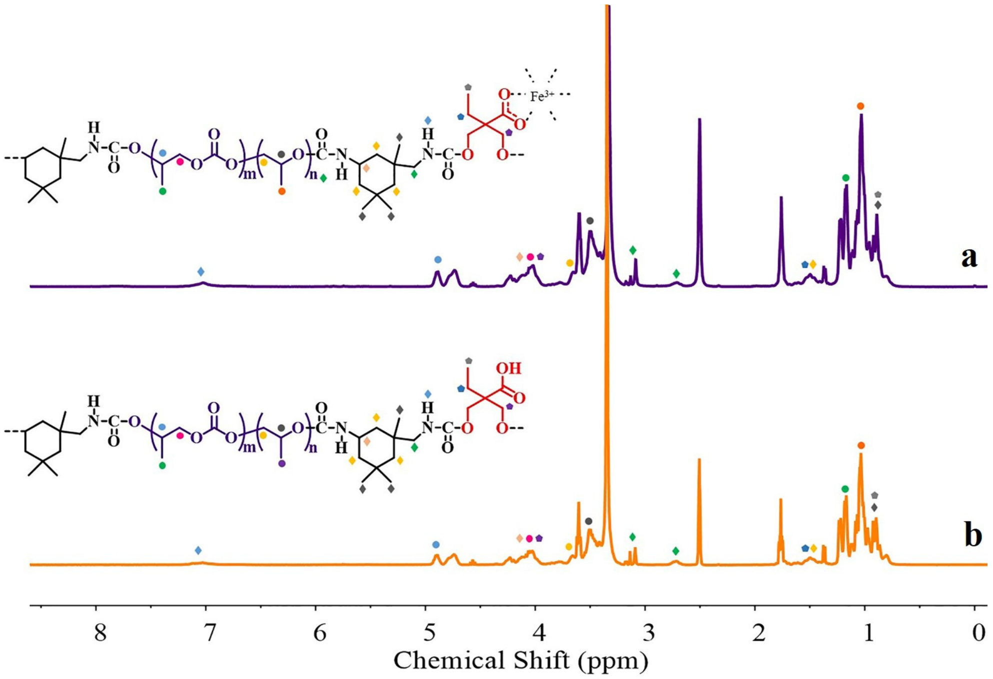

Figure S2. ^{1}H NMR spectra of (a) 0/18 Fe-TPU-Prepolymer and (b) 3/18 Fe-TPU-Prepolymer.

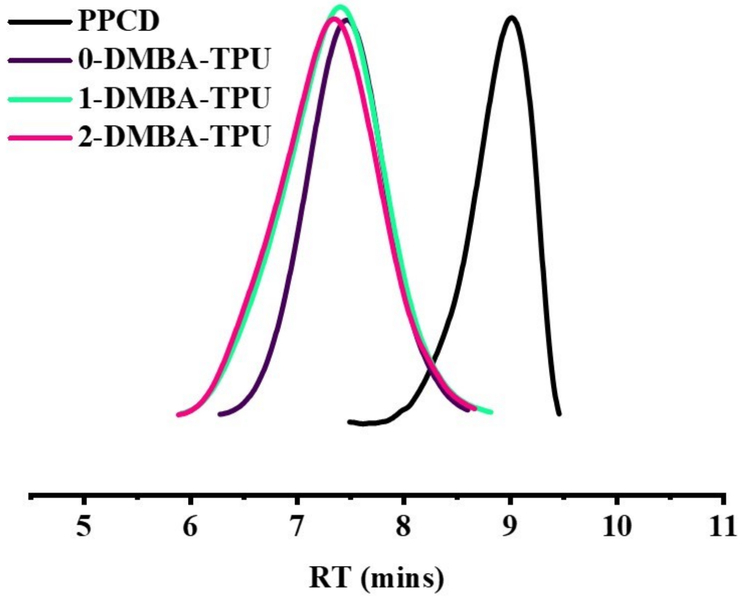

Figure S3. GPC curves of TPU with different DMBA contents.

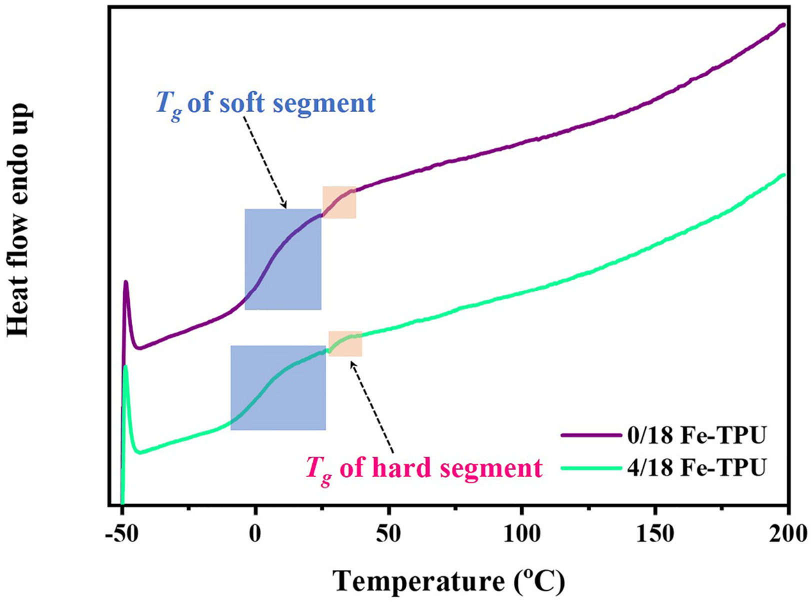

Figure S4. DSC curves of TPU reinforced with $FeCl_3$.

Table S2. DSC data of TPU reinforced with $FeCl_3$.

Entry	Sample	$T_{g,1}$ (°C)	$T_{g,2}$ (°C)
1	0/18 Fe-TPU	3.8	30.4
5	4/18 Fe-TPU	3.5	31.0

Entry 1 and Entry 5 belong to Table 2; $T_{g,1}$ and $T_{g,2}$ correspond to T_g of soft segment and hard segment respectively.
